# Survival Outcomes with Regorafenib and/or Trifluridine/Tipiracil Sequencing to Rechallenge with Third-Line Regimens in Metastatic Colorectal Cancer: A Multicenter Retrospective Real-World Subgroup Comparison from the ReTrITA Study

**DOI:** 10.3390/curroncol31120574

**Published:** 2024-12-04

**Authors:** Carlo Signorelli, Maria Alessandra Calegari, Annunziato Anghelone, Alessandro Passardi, Giovanni Luca Frassineti, Alessandro Bittoni, Jessica Lucchetti, Lorenzo Angotti, Emanuela Di Giacomo, Ina Valeria Zurlo, Cristina Morelli, Emanuela Dell’Aquila, Adele Artemi, Donatello Gemma, Domenico Cristiano Corsi, Alessandra Emiliani, Marta Ribelli, Federica Mazzuca, Giulia Arrivi, Federica Zoratto, Mario Giovanni Chilelli, Marta Schirripa, Maria Grazia Morandi, Fiorenza Santamaria, Manuela Dettori, Antonella Cosimati, Rosa Saltarelli, Alessandro Minelli, Emanuela Lucci-Cordisco, Michele Basso

**Affiliations:** 1 Medical Oncology Unit, Belcolle Hospital, ASL Viterbo, 01100 Viterbo, Italy; 2 Oncologia Medica, Comprehensive Cancer Center, Fondazione Policlinico Universitario Agostino Gemelli—IRCCS, 00168 Rome, Italy; 3 Department of Medical Oncology, IRCCS Istituto Romagnolo per lo Studio dei Tumori (IRST) “Dino Amadori”, 47014 Meldola, Italy; 4 Division of Medical Oncology, Policlinico Universitario Campus Bio-Medico, 00128 Rome, Italy; 5 Medical Oncology, “Vito Fazzi” Hospital, 73100 Lecce, Italy; 6 Medical Oncology Unit, Department of Systems Medicine, Tor Vergata University Hospital, 00133 Rome, Italy; 7 IRCCS Regina Elena National Cancer Institute, 00144 Rome, Italy; 8 Medical Oncology Unit, ASL Frosinone, 03039 Sora, Italy; 9 Medical Oncology, Isola Tiberina Hospital, Gemelli Isola, 00186 Rome, Italy; 10 Oncology Unit, Department of Clinical and Molecular Medicine, Sant’ Andrea University Hospital, Sapienza University of Rome, 00189 Rome, Italy; 11 UOC Oncologia, Ospedale Santa Maria Goretti, ASL Latina, 04100 Latina, Italy; 12 Medical Oncology Unit, San Camillo de Lellis Hospital, ASL Rieti, 02100 Rieti, Italy; 13 UOC Oncology A, Policlinico Umberto I, 00161 Rome, Italy; 14 Experimental Medicine, Network Oncology and Precision Medicine, Department of Experimental Medicine, Sapienza University of Rome, 00185 Rome, Italy; 15 Medical Oncology Department, Ospedale Oncologico Armando Businco, 09121 Cagliari, Italy; 16 Medical Oncology Department, UO Oncologia Universitaria della Casa della Salute di Aprilia, 04011 Aprilia, Italy; 17 UOC Oncology, San Giovanni Evangelista Hospital, ASL RM5, 00019 Tivoli, Italy; 18 Medical Oncology Department, UO Oncologia, Ospedale San Paolo, ASL RM4, 00053 Civitavecchia, Italy; 19 UOC Genetica Medica, Dipartimento di Scienze della Vita e Sanità Pubblica, Fondazione Policlinico Universitario Agostino Gemelli, IRCCS, 00168 Rome, Italy; 20 Medical Oncology Department, Comprehensive Cancer Center, Fondazione Policlinico Universitario Agostino Gemelli, IRCCS, 00168 Rome, Italy

**Keywords:** metastatic colorectal cancer, rechallenge therapy, regorafenib, trifluridine/tipiracil, third-line therapy, real-world study

## Abstract

Background: There is ongoing discussion around the optimal course of treatment for metastatic colorectal cancer (mCRC) following the second line. Trifluridine/tipiracil (T) and regorafenib (R) have been the mainstay of therapy in this situation, as they both increased overall survival (OS) in comparison to a placebo. Despite the paucity of evidence, therapy rechallenge is also recognized as an option for practical use. In the third-line scenario of mCRC, we planned to investigate the survival outcomes using (T) and (R), both with and without prior rechallenge treatment. Materials and methods: Between 2012 and 2023, we examined the medical records of 1156 patients with refractory mCRC who were enrolled in the multicenter retrospective ReTrITA study. We then separated the patients into two cohorts based on the rechallenge therapy that was given before regorafenib and/or trifluridine/tipiracil at 17 Italian centres. Results: A total of 981 patients underwent T and/or R therapy, while 175 patients had therapy rechallenge before T and/or R. The median overall survival (mOS) for patients treated with T/R and R/T sequences in the rechallenge therapy cohort was 14.5 months and 17.6 months, respectively (*p* = 0.1955). A statistically significant survival benefit was observed in patients who received monotheraphy with R (mOS: 6 months) compared to the T group (mOS: 4.2 months) (*p* = 0.0332). In the same cohort, a median progression-free survival (mPFS) benefit was demonstrated in favour of the R/T group (11.3 months) vs. 9 months of the reverse sequence (*p* = 0.4004). In the no-rechallenge cohort, the mOS was statistically longer in the R/T sequence than in the T/R sequence (16.2 months vs. 12.3 months, respectively; *p* = 0.0014). In terms of the mPFS, we saw the same significant result for the adoption of R/T treatment (11.5 months vs. 8.4 months, respectively; *p* < 0.0001). The two monotherapy groups did not reveal any significant differences. Conclusions: This study suggests that rechallenge therapy may improve survival rates in the third-line treatment of mCRC, particularly if it is administered before sequential R/T treatment. This could allow for the extension of mCRC treatment choices until prospective studies are finished or randomised trials are performed.

## 1. Introduction

Colorectal cancer (CRC) is the fourth most frequently diagnosed cancer and the second most common cause of cancer-related death in the United States. There is expected to be 106590 new cases of colon cancer and 46,220 cases of rectal cancer in 2024. Colon and rectal cancer will kill an estimated 53010 people this year [1,2]. Colorectal metastases occur in around 50% to 60% of CRC patients, and 80% to 90% of these people have unresectable metastatic liver disease [3,4,5,6]. Synchronous liver metastases occur in 20% to 34% of CRC patients [7].

There has been little progress in developing new treatments, and the standard of care in first- and second-line settings is still chemotherapy combined with oxaliplatin, irinotecan, and fluoropyrimidines. Sequencing thus becomes difficult and treatments are not as well defined after the third-line scenario. The usual third-line treatment is trifluridine/tipiracil (T) plus bevacizumab, according to the recently released SUNLIGHT trial results [8]. However, there are currently no long-term results or real-world data available. Beyond third-line therapy, alternative options, including regorafenib (R), anti-EGFR rechallenge, and chemotherapy retreatment, together with innovative therapies like anti-HER2 blocking, must be taken into consideration [9,10,11,12,13,14,15,16,17]. These options signal the beginning of a new era in colorectal cancer treatment.

To date, it is still unclear whether R or T is the preferable treatment strategy to start within clinical practice. In cases of wild-type RAS disease where the patient has not been previously exposed to such agents, as well as in cases of wild-type RAS/BRAF disease, anti-EGFRs have been shown to be a viable treatment option [18,19,20,21]. Currently, in actual clinical settings, rechallenge therapy, which is the administration of chemotherapy regimens and biologic agents to which patients have previously been exposed, is frequently taken into consideration after standard therapies have failed and because novel therapeutic approaches such as immunotherapy and targeted agents have not shown efficacy in this context, with the exception of small genomically selected subpopulations [22]. The underlying theory behind this approach is that tumour cells might become more sensitive or remain sensitive to chemotherapy following the discontinuation of treatment, which could have positive clinical effects [23,24,25,26,27,28,29,30,31,32,33]. That being said, there is still little proof to back up this approach. Though these treatments are available, there are not much data that compare the effectiveness of chemotherapy retreatment, R, and T in the third-line treatment of metastatic colorectal cancer [34,35].

Trifluridine, a nucleoside analogue, and tipiracil, a thymidine phosphorylase inhibitor, are taken orally together. Trifluridine has been demonstrated to accumulate intracellularly in response to this combination, which inhibits DNA synthesis and ultimately results in cell death. In patients with mCRC who had progressed on traditional treatments, the RECOURSE trial showed a substantial improvement in their OS with T when compared with a placebo [10]. Conversely, the oral multikinase inhibitor R targets several signalling pathways linked to tumour growth, angiogenesis, and metastasis. In patients with mCRC who had progressed on conventional treatments, the CORRECT trial demonstrated a statistically significant improvement in OS with R when compared with a placebo [11]. For the management of mCRC, there is an urgent need for new active agents as well as the best possible use of treatments already in clinical use.

To further explain whether, in real-world practice, retreatment therapy is truly superior in terms of survival compared with standard third-line therapy with T and R, we conducted this subgroup analysis from the multicenter retrospective ReTrITA study [36,37].

## 2. Patients and Methods

The current analysis was conducted using data from the soon-to-be-published observational, retrospective, multicenter ReTrITA study. The purpose of the study was to evaluate the survival outcomes of R and T treatment when used either sequentially or as monotherapy in patients with mCRC after standard treatments failed. The study received approval by the Ethics Committee of Area 4 Lazio, Rome, Italy, with protocol number 29-2024, 4 March 2024.

### 2.1. Study Design

In this retrospective substudy, we focused on the predictive survival significance of rechallenge therapy in patients treated with R or T, sequentially or in monotherapy. Rechallenge therapy was described as reusing the same or a similar 5-fluouracil (5-FU)-based regimen that was administered in one of the first two lines of mCRC. As a challenge, 5-FU-based chemotherapy was given with either oxaliplatin (FOLFOX/XELOX), irinotecan (FOLFIRI/XELIRI), or both (FOLFIRINOX). Fluoropyrimidines were administered both orally and intravenously. All patients who experienced biological treatment consisting of either anti-EGFR antibody (cetuximab, panitumumab) or anti-VEGF antibody (bevacizumab, aflibercept) in combination with chemotherapy based on their KRAS, NRAS, and BRAF mutational status, were enrolled. If patients were given maintenance therapy after establishing disease control, induction followed by maintenance treatment would constitute one line of therapy. Patients who administered a first-line irinotecan-containing regimen were permitted to receive a single-agent irinotecan as the third line.

**Figure 1:**
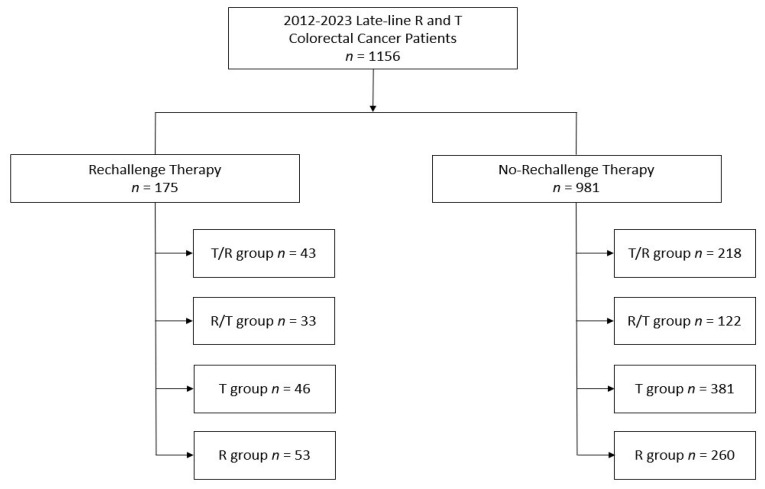
Study design.

Depending on whether the patients received rechallenge treatment as third-line therapy, they were divided into two cohorts. After that, each cohort was further divided up into smaller groups in order to compare the survival results obtained from standard third-line treatment with R and T versus rechallenge therapy followed by R and T. Both sequential administration and monotherapy were evaluated for the latter. Patients were followed until they died or lost contact. The study design is shown in Figure 1.

### 2.2. Outcomes Parameters

The primary endpoints of this analysis were overall survival (OS), which is the time interval between the start of R and/or T treatment and death from any cause (in the sequential treatment groups, OS was defined as the time between the first treatment with R or T and death during the second treatment with T or R), and progression-free survival (PFS), which is the time interval between the start of treatment with R and/or T and disease progression or death (in the sequential treatment groups, PFS was defined as the time between the first treatment with T or R and disease progression or death during the second treatment with R or T). During the most recent follow-up, patients who did not experience an event were excluded.

The secondary objective was to identify potential response variables that could predict survival. To reduce the potential for selection bias, all patients who received R and/or T, either alone or sequentially, were included in the present study. The patients were selected by a designated investigator who was unaware of the findings and conclusions of the study. The lead investigator, who was responsible for overseeing the statistical analyses, was not involved in the selection of patients. At the time of the investigation, endpoints were established in order to reduce the likelihood of distortion bias. Given the retrospective nature of the study, it is important to recognise that the findings should be regarded as exploratory. The confidentiality of the patient data was maintained and, in accordance with the retrospective nature of the study, the requirement for informed consent was waived in cases where the patient was unreachable and/or unable to provide consent.

### 2.3. Drugs Administration

Oral trifluridine/tipiracil was administered twice daily at a dose of 35 mg/m^2^ on days 1–5 and 8–12, with two days off, for a duration of two weeks. At the conclusion of each month, there was a 14-day rest period. A normal dose of 160 mg of regorafenib was administered once daily for 21 days throughout a 28-day cycle. The doctor decided to adopt the ReDos dose-escalation method of R, which starts with an oral dose of 80 mg/day and increases every week by 40 mg increments up to 160 mg/day if no substantial drug-related side effects occur [38]. Every treatment was administered in compliance with the Declaration of Helsinki and continued until the disease progressed, unacceptable side effects occurred, or until the investigator decided whether it was thought to be clinically required.

### 2.4. Statistical Analysis

Utilising descriptive statistics, pertinent data were gathered. Potential relationships were assessed using the Fisher exact test and the Chi-square test. Using the Kaplan–Meier product limit method, PFS and OS were estimated and the log-rank test was used to evaluate subgroup differences. The significance level was set at *p* ≤ 0.05. All statistical analyses were performed using SPSS Statistics software, version 21.0. When plotting survival estimates for the treatment groups using the Kaplan–Meier method, an exploratory subgroup analysis was carried out to compare OS and PFS between the treatment groups, stratified by age, sex, ECOG performance status (PS), RAS status, and metastatic disease sites.

## 3. Results

### 3.1. Patient Characteristics

From the original ReTrITA study, we conducted a retrospective analysis to identify patients with mCRC who had been treated with T or R at 17 Italian centres between 2012 and 2023. Of the 1156 patients eligible for inclusion in the study, 261, 155, 427, and 313 were assigned to the T/R, R/T, T, and R groups, respectively. In the whole study population, the median follow-up duration was 7.6 months (95% CI = 7.1 to 101). In the group receiving rechallenge therapy, this was 8.5 months (95% CI = 7.3 to 71.9), whereas in the group not receiving rechallenge therapy, it was 7.3 months (95% CI = 7 to 101). In the present analysis, we concentrated our attention on the 175 patients who received rechallenge therapy prior to R and/or T treatment (15.1%) in comparison with the 981 patients (84.9%) who were in the R and/or T groups and did not receive prior rechallenge therapy. A comparison of baseline characteristics of the rechallenge and no-rechallenge groups is shown in Table 1.

The median age was higher in patients who received T treatment and had not undergone prior rechallenge (71 years old). The majority of patients in both groups were male. With regard to the primary tumor location, 45.9% of the patients had left-sided colon cancer, 33% had right-sided colon cancer, and 21.1% had rectal cancer, unaffected by the administered treatment. The study population included a greater proportion of patients younger than 70 years, particularly in the R group (66% after rechallenge therapy). With regard to RAS status, the majority of patients with mutant RAS were in the T-treated patients with no-rechallenge group (66.1%), whereas 73.6% of R-treated patients harbored wild-type RAS after rechallenge. A total of 9.3% of patients in the T/R group after rechallenge therapy exhibited dMMR (mismatch repair gene deficiency).

In the rechallenge cohort, 44 patients (25.1%) received oxaliplatin-FU-based chemotherapy, such as FOLFOX or XELOX. FOLFIRI or XELIRI were administered to 52 patients (29.7%), with 21 receiving anti-VEGF treatment, 15 receiving anti-EGFR treatment, and 16 receiving neither. FOLFOXIRI was administered to two patients (1.1%), one in combination with anti-VEGF treatment and the other with anti-EGFR treatment. Other treatments included capecitabine monotherapy (22 patients, 12.6%), 5-FU/Leucovorin infusion (10 patients, 5.7%), irinotecan (23 patients,13.1%), and anti-EGFR monotherapy (22 patients, 12.6%).

### 3.2. Survival Outcomes in the Rechallenge Cohort

**Figure 2:**
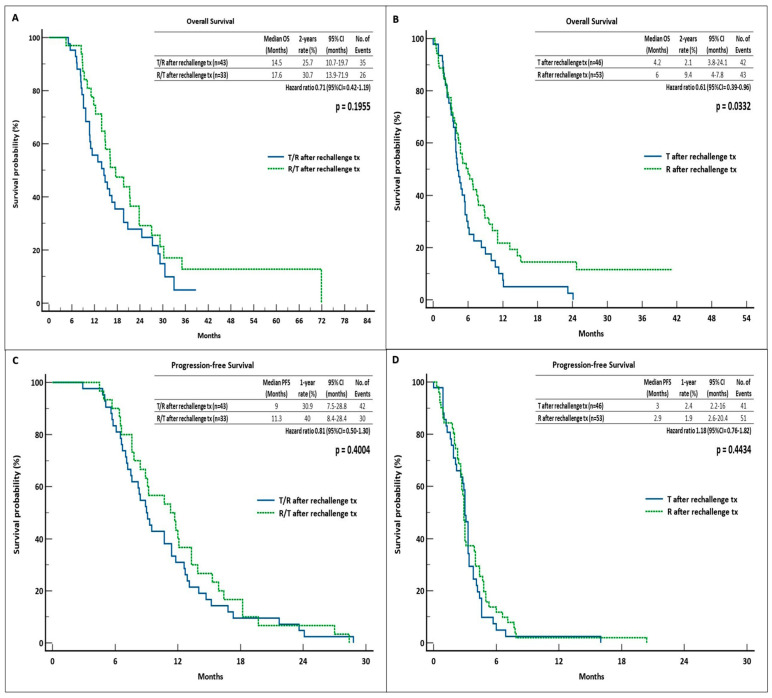
Survival outcomes in the rechallenge cohort. (**A**) OS in T/R and R/T groups; (**B**) OS in T and R groups; (**C**) PFS in T/R and R/T groups; (**D**) PFS in T and R groups. Abbreviations: OS, overall survival; PFS, progression-free survival; tx, therapy; CI, confidence interval; R, regorafenib; T, trifluridine/tipiracil.

In this cohort, the mOS and 2-year OS rates were 14.5 months (95% CI = 10.7–19.7) and 25.7% versus 17.6 months (95% = 13.9–71.9) and 30.7% for patients treated with T/R and R/T sequences, respectively [hazard ratio (HR) = 0.71, log-rank *p* = 0.1955)]. Regarding mPFS, a benefit was shown in favor of R/T group (11.3 months and 1-year rate = 40%, 95% CI = 8.4–28.4) compared to 9 months and 30.9%, respectively, for the inverse sequence (95% CI = 7.5–28,8) (HR = 0.81, log-rank *p* = 0.4004). On the other hand, we observed a statistically significant survival benefit in patients who received monotheraphy with R (mOS: 6 months, 95% CI = 4.0–7.8, 2-year OS rate = 9.4%) versus the T group (mOS: 4.2 months, 95% CI = 3.8–24.1, 2-year OS rate = 2.1%]) (HR = 0.61, log-rank *p* = 0.0332). With regard to PFS, no notable discrepancies were identified between the two monotherapy groups (mPFS: 3 months, 95% CI = 2.2–16, 1-year PFS rate = 2.4% for the patients treated with T) (mPFS: 2.9 months, 95% CI = 2.6–20.4, 1-year PFS rate = 1.9% for the R patients) (HR = 1.18, log-rank *p* = 0.4434) (Figure 2).

### 3.3. Survival in the No-Rechallenge Cohort

**Figure 3:**
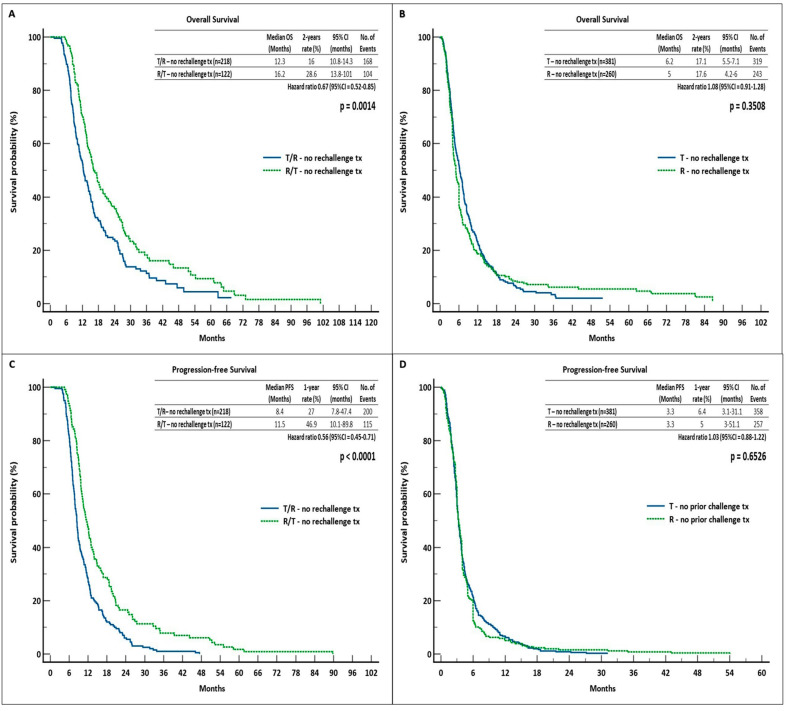
Survival outcomes in the no-rechallenge cohort. (**A**) OS in T/R and R/T groups, (**B**) OS in T and R groups, (**C**) PFS in T/R and R/T groups, (**D**) PFS in T and R groups. Abbreviations: OS, overall survival; PFS, progression-free survival; tx, therapy; CI, confidence interval; R, regorafenib; T, trifluridine/tipiracil.

We found that administering R before T significantly improved patients’ chances of survival in those who did not receive rechallenge as third-line therapy. Specifically, the mOS and 2-year OS rate were longer in the R/T sequence group than in the T/R sequence group (16.2 months and 28.6%, 95% CI = 13.8–101 vs. 12.3 months and 16%, 95% CI = 10.8–14.3, respectively) (HR = 0.67, log-rank *p* = 0.0014). In terms of the mPFS, we observed a similar outcome for the use of R/T treatment. Further analysis revealed that the R/T group reported mPFS and 1-year PFS rates that were statistically longer than T/R-treated patients (11.5 months and 46.9%, 95% CI = 10.1–89.8 compared to 8.4 months and 27%, 95% CI = 7.8–47.4, respectively) (HR = 0.56; log-rank *p* < 0.0001). The two monotherapy groups failed to show any noticeable differences; the two-year OS and mOS rates, with a log-rank *p* = 0.3508, were 6.2 months and 17.1% in the T group (95% CI = 5.5–7.1) and 5 months and 17.6% in the R-treated patients (95% CI = 4.2–6) (HR = 1.08). PFS differences were likewise not statistically significant. In fact, with a log-rank *p* = 0.6526, we found that the groups reported the same mPFS of 3.3 months (1-year PFS = 6.4%, 95% CI = 3.1–31.1 vs. 1-year PFS rate = 5%, 95% CI = 3–54.1, HR = 1.03) (Figure 3).

### 3.4. Comparison of Survival Results Between the 2 Cohorts

**Figure 4:**
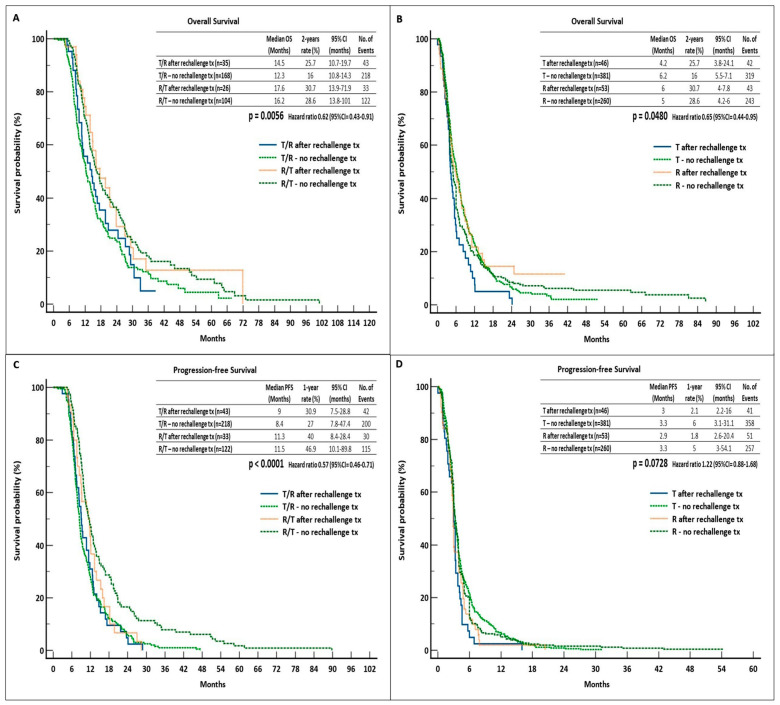
Survival outcomes in the rechallenge cohort vs. the no-rechallenge cohort. (**A**) OS in T/R and R/T groups, (**B**) OS in T and R groups, (**C**) PFS in T/R and R/T groups, (**D**) PFS in T and R groups. Abbreviations: OS, overall survival; PFS, progression-free survival; tx, therapy; CI, confidence interval; R, regorafenib; T, trifluridine/tipiracil.

We compared the survival outcomes of the two cohorts and found that patients who received R/T after rechallenge therapy had significantly longer mOS and the best two-year OS rate vs. the other groups (17.6 months and 30.7%, HR = 0.62, interaction *p* = 0.0056). Both the cohort receiving the rechallenge therapy (11.3 months) and the cohort that did not receive it (11.5 months) showed a statistically significant longer mPFS associated with R/T (40% and 46.9% 1-year rate, respectively; HR = 0.57, interaction *p* < 0.0001). Regarding mOS in the monotherapy groups, there was no noticeable disparity between the administration of R after rechallenge therapy and T without it (6.2 vs. 6 months but different 2-year rates of 17.1 vs. 9.4%, respectively) (HR = 0.65, interaction *p* = 0.0480). Lastly, there were no PFS advantages in the monotherapy groups (3.3 months of both T and R without rechallenge therapy vs. 3 and 2.9 months of T and R after rechallenge therapy, respectively; HR = 1.22, interaction *p* = 0.0728) (Figure 4).

### 3.5. Subgroups Survival Comparative Analysis

**Figure 5:**
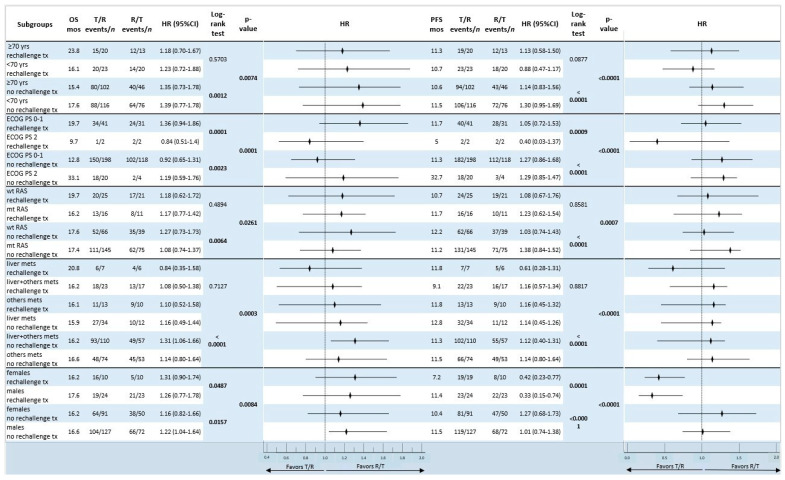
Forest plot of subgroups overall survival and progression-free survival analysis according to baseline characteristics of patients who received sequential treatment with trifluridine/tipiracil and regorafenib in the rechallenge cohort vs. the no-rechallenge cohort. Statistically significant *p*-values are reported in bold. Abbreviations: OS, overall survival; PFS, progression-free survival; mos, months; HR, hazard ratio; PS, performance status; T, trifluridine/tipiracil; R, regorafenib; mets, metastases; wt, wild type; mt, mutant type; CI, confidence interval; yrs, years; *n*, number.

An exploratory subgroup analysis comparing the survival outcomes between the two cohorts based on baseline characteristics and the R and T administration schedule showed that in the sequential treatment, there was a correlation between the R/T sequence and longer mOS in patients who were ≥ 70 years old (23.8 months after rechallenge therapy, *p* = 0.0074), had an ECOG PS = 2 (33.1 months with no rechallenge therapy, *p* = 0.0001), in those with wild-type RAS (19.7 months, *p* = 0.0261), and in men (17.6 months after rechallenge therapy, *p* = 0.0084). In this particular context, a longer mOS was observed in patients who had liver metastases only (20.8 months after rechallenge therapy, *p* = 0.0003). Within the sequence groups, patients with an ECOG PS = 2 (32.7 months with R/T without rechallenge therapy administration, *p* < 0.0001), patients with liver metastases only (12.8 months with R/T and without rechallenge therapy, *p* < 0.0001), and men who received rechallenge therapy and subsequent T/R sequence (11.4 months, *p* < 0.0001) showed a significant benefit in terms of the mPFS (Figure 5).

**Figure 6:**
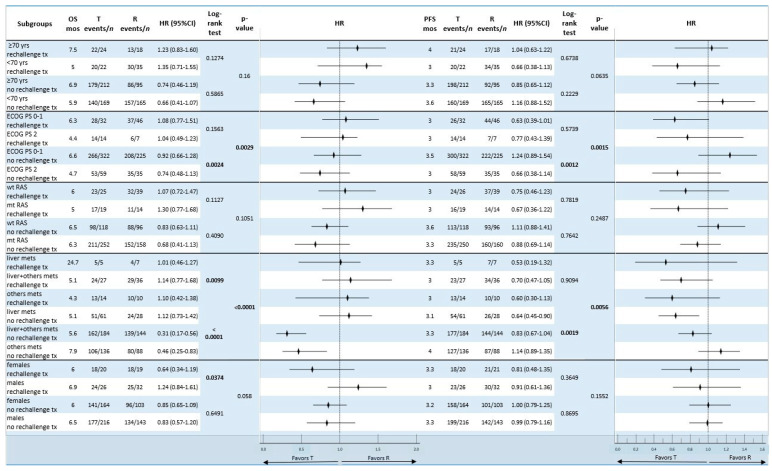
Forest plot of subgroups’ overall survival and progression-free survival analysis according to baseline characteristics of patients who received monotherapy with trifluridine/tipiracil and regorafenib in the rechallenge cohort vs. the no-rechallenge cohort. Statistically significant *p*-values are reported in bold. Abbreviations: OS, overall survival; PFS, progression-free survival; mos, months; HR, hazard ratio; PS, performance status; T, trifluridine/tipiracil; R, regorafenib; mets, metastases; wt, wild type; mt, mutant type; CI, confidence interval; yrs, years; *n*, number.

We did not find any noteworthy differences in survival between subgroups for the patients who received T or R monotherapy, with the exception of T in patients with an ECOG PS = 0–1 (mOS = 6.6 months without rechallenge therapy, *p* = 0.0029) and those with liver metastases only (mOS = 24.7 months after rechallenge therapy). Regarding the monotherapy groups, no significant differences in mPFS were found (Figure 6).

## 4. Discussion

Rechallenge therapy may be an attractive treatment choice for patients who qualify for conventional chemotherapy when other treatment options have restrictions, taking into account performance status, previous treatment response, and, in particular, persistent toxicities. Patients should be carefully selected, especially before applying the rechallenge strategy at the third stage, based on their current disease burden and residual toxicity. In four randomized trials [10,11,12,39], R and T were compared separately to the best supportive care in patients with mCRC who had failed at least two lines of therapy. The outcomes demonstrated statistically significant improvements in PFS and OS. The results of these investigations led to the approval of the two agents by the Food and Drug Administration and the European Medicines Agency, and they are currently the preferred third-line options in many clinical practice guidelines. The recent SUNLIGHT trial demonstrated that the combination of T plus bevacizumab was more efficacious than T alone, with increased PFS and OS [8]. It is regrettable that the aforementioned combination was not the standard of care at the time our data were collected. Consequently, a re-evaluation is required to enable the comparison of a combination of T and bevacizumab with chemotherapy rechallenge. Furthermore, there is only one prospective head-to-head trial that has directly assessed sequential R and T, the PRODIGE 68-UCGI 38-SOREGATT study, which was prematurely ended due to the publication of data from the SUNLIGHT study [40]. It is still unclear which is the preferable treatment strategy to start with, either R, T, or T + BEV, in clinical practice and whether there exists an ideal sequence for these two drugs. There were two abstracts given at the 2024 American Society of Clinical Oncology Gastrointestinal Cancer Symposium. The first was the OSERO study, an observational study that examined and monitored R and T in a Japanese patient group. The mPFS was 7.1 months for T, approximately 12 months for R, and 10.3 months for T plus bevacizumab [41]. It is important to note that this was an Asian patient population. The study included a fairly large number of approximately 450 patients who were examined on this. The second is from the database of Flatiron Health, from a retrospective cohort that questioned individuals who had care from 2015 to 2023 about the same thing [42]. Some patients had T after R, whereas others received R first, then T. This was prior to the availability of fruquintinib data. When R was followed by T, the median OS was 13.1 months; when T was followed by R, the median OS was 11.5 months (adjusted HR, 0.99; 95% CI, 0.75–1.29). It was comparable but not statistically significant. Neither of these studies provided us with any evidence that one was better than the other. However, the comparison of R and T with rechallenge therapy has not been the subject of a randomised trial.

Against this background, we aimed to perform this real-world analysis based on observations in clinical practice of subgroups from the soon-to-be-published retrospective ReTrITA study. Our aim was to investigate whether rechallenge treatment followed by standard treatment with R or T either as a monotherapy or sequentially, compared with standard treatment at the time of data collection (R or T as a monotherapy or sequentially), would have an impact on survival outcomes. Our analysis of a larger population of 1156 patients revealed that in cases of molecularly unselected, refractory mCRC, the R/T therapy sequence administered after third-line rechallenge therapy resulted in a significant increase in overall survival (17.6 months) compared to the reverse T/R sequence (14.5 months). The difference is especially noticeable when two groups of patients who had sequential R and T therapy without preceding rechallenge therapy were directly compared (16.2 months in the R/T group vs. 12.3 months in the T/R group) (log-rank *p* = 0.0014). The R/T sequence still has a considerable advantage in terms of mPFS (11.3 vs. 11.5 months, *p* < 0.0001), regardless of rechallenge therapy. The no-rechallenge cohort’s mOS for patients who received monotherapy with either R or T was comparable to that shown in clinical trials that resulted in the FDA approvals (5 and 6.2 months, respectively). This implies that in a real-world population, the small advantage of these agents is not diminished. Our investigation could add important information to the literature without prospectively randomised trials comparing the rechallenge treatment with R and T in refractory mCRC.

After reviewing the literature, we identified four retrospective studies that aimed to assess the effectiveness of rechallenge therapy using either T or R. Kostek conducted research in which 104 mCRC patients who had not responded to two lines of treatment were retrospectively assessed based on the type of third-line treatment they had received. When comparing the rechallenge group to the R group, the median PFS was 9.16 months versus 3.41 months (HR = 0.22, *p* < 0.001). For the rechallenge group, the OS was 12.0 months, but for the R group, it was 6.6 months (HR = 0.29, *p* < 0.001). Tasci et al. compared 128 patients who received rechallenge therapy with 266 patients who received R therapy. The two groups’ PFS rates were 5.82 and 4 months, respectively (HR = 1.45, *p* = 0.167), and the OS rates of the rechallenge and R groups were significantly higher (11.99 months vs. 8.8 months; HR = 1.51, *p* < 0.001). Caligari et al. found that patients treated with chemotherapy rechallenge or reintroduction (CTr/r) had a significantly longer mOS than those treated with R or T in the PROSERpINa study (18.5 months for CTr/r, 6 months for R, and 7.6 months for T, *p* < 0.0001). The mPFS rates for patients treated with CTr/r, T, and R were 6.1, 3.9, and 3 months, respectively (*p* < 0.0001). Notably, the authors of this study conducted a propensity score analysis that took into account the number of metastatic sites, the treatment line, and ECOG PS. In the latest study, Bazarbashi et al. found that the chemotherapy rechallenge group had a significant difference in OS (21.2 vs. 12.6 months, *p* = 0.006), but the PFS for the rechallenge group was not better than that of the T/R group (3.1 vs. 2.9 months, respectively, *p* = 0.357).

There is a strong need to discover relevant clinical traits or biomarkers that can predict survival outcomes. We performed several subgroup analyses to identify potential prognostic markers. In the rechallenge cohort, OS and PFS were both significantly better in the R/T groups, whereas the OS was best in the R-treated groups. In the no-rechallenge cohort, the R/T sequence was confirmed as the best in terms of both OS and PFS, while in the monotherapy groups T was predominant in terms of the survival results. In detail, in the cohort undergoing rechallenge therapy, we found a statistical trend towards better prognosis with R/T sequential therapy than with T/R sequence in patients with an ECOG PS = 0–1, in men, and with R monotherapy compared to T in patients with only hepatic metastases, especially in men. In the cohort that did not undergo rechallenge therapy, the R/T therapeutic sequence was statistically superior in prognostic terms in patients who were under 70 years of age, had an ECOG PS = 2, had RAS wt tumors with only hepatic metastases, and in men, while T brought significantly better results in patients with ECOG PS = 1 and in patients with metastases that were not hepatic. Therefore, we could hypothesise that for some pretreated patients, rechallenge therapy before the R/T sequence approach might be taken into consideration. Our findings could be very interesting with regard to the continuum of care for patients with mCRC because they answer a variety of questions that arise in daily clinical practice and suggest what to do or not do after rechallenge therapy, particularly in patients with an ECOG PS = 2, with different metastatic patterns, or in males versus females, among other factors. It would be justified and desirable to confirm our findings by conducting prospective trials with randomised controls.

One of our study’s main advantages is that it involves a population of patients that is typical of those seen in daily clinical settings, so our analysis is more clinically applicable as a result. The main limitation of this research is its retrospective design, which could have resulted in bias because the study treatments were not randomized, although it is possible that the medical records collected at the several institutes might be biased. The numerical imbalance between the rechallenge and control cohorts and the small sample sizes of some subgroups, such as those with an ECOG PS = 2, may have affected the reliability of the analysis. Furthermore, the effect of locally ablative and surgical treatments—which are usually saved for subsequent therapeutic modalities—was not evaluated. Rechallenge therapy, however, is a promising third-line option when considering the effectiveness of R and T therapy in patients who become resistant after multiple lines of therapy. This provides a way to extend the mCRC treatment options until prospective studies are completed or randomised trials are designed.

## 5. Conclusions

The purpose of this study was to determine which of the two treatment modalities—trifluridine/tipiracil and regorafenib, with or without preceding rechallenge therapy—is more effective when administered as a third or fourth treatment for advanced refractory colorectal cancer. The results indicate that patients who received trifluridine/tipiracil after regorafenib with previous rechallenge therapy had a significantly longer survival time and remained on treatment for longer than those who received the two drugs in the opposite order. This was especially true for male patients and those whose ECOG PS was 0–1. Following rechallenge therapy, no noteworthy outcomes were observed in the monotherapy with R or T groups. Even though the results are limited by the small numbers in certain patient subgroups, they offer insight into the possible outcomes, particularly when patients receive sequential R/T following rechallenge therapy.

## Figures and Tables

**Table 1:** Patients’ characteristics at baseline.

Variables	Rechallenge Therapy Group					No-Rechallenge Therapy Group				
	T/R	R/T	T	R	*p*-Value	T/R	R/T	T	R	*p*-Value
	No. (%)	No. (%)	No. (%)	No. (%)		No. (%)	No. (%)	No. (%)	No. (%)	
**No. of patients**	43 (100)	33 (100)	46 (100)	53 (100)	0.19	218 (100)	122 (100)	381 (100)	260 (100)	<0.0001
**1°line therapy**										
Single agent	2 (4.7)	-	4 (8.7)	1 (1.9)	0.21	16 (7.6)	6 (5.0)	33 (8.8)	8 (3.1)	<0.0001
Doublet CT	37 (86.0)	27 (81.8)	36 (78.3)	49 (92.5)		178 (84.8)	101 (83.5)	307 (82.3)	119 (45.8)	
Triplet CT	4 (9.3)	6 (18.2)	6 (13.0)	3 (5.7)		16 (7.6)	7 (5.8)	32 (8.6)	14 (5.4)	
Unknown	-	-	-	-		-	7 (5.8)	1 (0.3)	119 (45.8)	
Anti-EGFR use	21 (56.8)	12 (46.2)	13 (37.1)	22 (45.8)	0.42	55 (30.9)	32 (35.2)	85 (32.1)	40 (39.6)	0.46
Anti-VEGF use	16 (43.2)	14 (53.8)	22 (62.9)	26 (54.2)		123 (69.1)	59 (64.8)	180 (67.9)	61 (60.4)	
**2°line therapy**										
Single agent	7 (16.3)	4 (12.1)	4 (8.7)	6 (11.3)	0.84	16 (7.7)	7 (5.8)	44 (12.0)	11 (4.3)	<0.0001
Doublet CT	34 (79.1)	27 (81.8)	39 (84.8)	46 (86.8)		167 (80.7)	97 (80.2)	284 (77.6)	105 (41.2)	
Triplet CT	2 (4.7)	2 (6.1)	3 (6.5)	1 (1.9)		4 (1.9)	2 (1.7)	5 (1.4)	6 (2.4)	
Unknown	-	-	-	-		20 (9.7)	15 (12.4)	33 (9.0)	133 (52.2)	
Anti-EGFR use	2 (6.1)	5 (16.1)	9 (26.5)	6 (15.8)	0.16	14 (9.6)	5 (5.5)	18 (7.3)	8 (8.3)	0.68
Anti-VEGF use	31 (93.9)	26 (83.9)	25 (73.5)	32 (84.2)		132 (90.4)	86 (94.5)	230 (92.7)	88 (91.7)	
**3°line regimen**										
Trifluridine/tipiracil	-	-	-	-	0.43	218 (100)	-	381 (100)	-	<0.0001
Regorafenib	-	-	-	-		-	122 (100)	-	260 (100)	
Mono CT	5 (11.6)	2 (6.1)	3 (6.5)	8 (15.1)		-	-	-	-	
Mono CT + anti-EGFR	11 (25.6)	2 (6.1)	7 (15.2)	10 (18.9)		-	-	-	-	
Mono CT + anti-VEGF	1 (2.3)	3 (9.1)	2 (4.3)	1 (1.9)		-	-	-	-	
Doublet CT	13 (30.2)	9 (27.3)	14 (30.4)	13 (24.5)		-	-	-	-	
Doublet CT + anti-EGFR	5 (11.6)	5 (15.2)	4 (8.7)	6 (11.3)		-	-	-	-	
Doublet CT + anti-VEGF	3 (7.0)	5 (15.2)	11 (23.9)	8 (15.1)		-	-	-	-	
Triplet CT	-	-	-	-		-	-	-	-	
Triplet CT + anti-EGFR	1 (2.3)	-	-	-		-	-	-	-	
Triplet CT + anti-VEGF	1 (2.3)	-	-	-		-	-	-	-	
Anti-EGFR alone	3 (7.0)	7 (21.2)	5 (10.9)	7 (13.2)		-	-	-	-	
**Age Median (min–max)**	69 (30-83)	66 (47-86)	70 (47-87)	66 (45-86)	0.97	69 (40-86)	67 (43-85)	71 (42-88)	64 (34-86)	0.94
**Age**										
≥70 yrs	20 (46.5)	13 (39.4)	24 (52.2)	18 (34.0)	0.29	102 (46.8)	46 (37.7)	212 (55.6)	95 (36.5)	<0.0001
<70 yrs	23 (53.5)	20 (60.6)	22 (47.8)	35 (66.0)		116 (53.2)	76 (62.3)	169 (44.4)	165 (63.5)	
**Sex**										
Female	19 (44.2)	10 (30.3)	20 (43.5)	21 (39.6)	0.6	91 (41.7)	50 (41.0)	164 (43.0)	107 (41.2)	0.95
Male	24 (55.8)	23 (69.7)	26 (56.5)	32 (60.4)		127 (58.3)	72 (59.0)	217 (57.0)	153 (58.8)	
**RAS status**										
Wild type	25 (58.1)	21 (63.6)	26 (56.5)	39 (73.6)	0.48	66 (30.3)	39 (32.0)	118 (31.0)	96 (36.9)	0.09
Mutant type	16 (37.2)	11 (33.3)	19 (41.3)	14 (26.4)		141 (64.7)	75 (61.5)	252 (66.1)	160 (61.5)	
Unknown	2 (4.7)	1 (3.0)	1 (2.2)	-		11 (5.0)	8 (6.6)	11 (2.9)	4 (1.5)	
**Primary tumor location**										
Right side	13 (30.2)	7 (21.2)	14 30.4)	12 (22.6)	0.36	81 (37.2)	41 (33.6)	125 (32.8)	88 (33.8)	<0.0001
Left side	21 (48.8)	15 (45.5)	17 (37.0)	31 (58.5)		84 (38.5)	52 (42.6)	162 (42.5)	149 (57.3)	
Rectum	9 (20.9)	11 (33.3)	15 (32.6)	10 (18.9)		53 (24.3)	29 (23.8)	94 (24.7)	23 (8.8)	
**MSI**										
Yes	4 (9.3)	2 (6.1)	-	-	0.0007	4 (1.8)	6 (4.9)	6 (1.6)	2 (0.8)	<0.0001
No	29 (67.4)	19 (57.6)	16 (34.8)	25 47.2)		155 (71.1)	77 (63.1)	257 (67.5)	102 (39.2)	
Unknown	10 (23.3)	12 (36.4)	30 (65.2)	28 (52.8)		59 (27.1)	39 (32.0)	118 (31.0)	156 (60.0)	
**PS ECOG**										
0	20 (46.5)	14 (42.4)	6 (13.0)	15 (28.3)	0.0015	73 (33.5)	43 (35.2)	77 (20.2)	67 (25.8)	0.0001
1	21 (48.8)	16 (48.5)	26 (56.5)	31 (58.5)		125 (57.3)	75 (61.5)	245 (64.3)	158 (60.8)	
2	2 (4.7)	3 (9.1)	14 (30.4)	7 (13.2)		20 (9.2)	4 (3.3)	59 (15.5)	35 (13.5)	
**Prior adjuvant therapy**										
Yes	9 (20.9)	7 (21.2)	13 (28.3)	16 (30.2)	0.66	71 (32.6)	49 (40.2)	102 (26.8)	46 (17.7)	<0.0001
No	34 (79.1)	26 (78.8)	33 (71.7)	37 (69.8)		147 (67.4)	73 (59.8)	279 (73.2)	214 (82.3)	
**Metastatic disease sites**										
Liver only	7 (16.3)	6 (18.2)	5 (10.9)	7 (13.2)	0.63	34 (15.6)	12 (9.8)	61 (16.0)	28 (10.8)	0.16
Liver + other	23 (53.5)	17 (51.5)	26 (56.5)	36 (67.9)		109 (50.0)	57 (46.7)	184 (48.3)	144 (55.4)	
Others	13 (30.2)	10 (30.3)	15 (32.6)	10 (18.9)		75 (34.4)	53 (43.4)	136 (35.7)	88 (33.8)	

Abbreviations: T, trifluridine/tipiracil; R, regorafenib; MSI, micro-satellites’ instability; PS, performance status; yrs, years; CT, chemotherapy.

## Data Availability

The data to support the results reported in this study are available from the corresponding author on reasonable request.
